# Alternatives to the Six-Minute Walk Test in Pulmonary Arterial Hypertension

**DOI:** 10.1371/journal.pone.0103626

**Published:** 2014-08-11

**Authors:** Vincent Mainguy, Simon Malenfant, Anne-Sophie Neyron, Didier Saey, François Maltais, Sébastien Bonnet, Steeve Provencher

**Affiliations:** Pulmonary Hypertension Research Group, Centre de recherche de l'Institut universitaire de cardiologie et de pneumologie de Québec, Université Laval, Québec (Québec), Canada; Vanderbilt University Medical Center, United States of America

## Abstract

**Introduction:**

The physiological response during the endurance shuttle walk test (ESWT), the cycle endurance test (CET) and the incremental shuttle walk test (ISWT) remains unknown in PAH. We tested the hypothesis that endurance tests induce a near-maximal physiological demand comparable to incremental tests. We also hypothesized that differences in respiratory response during exercise would be related to the characteristics of the exercise tests.

**Methods:**

Within two weeks, twenty-one PAH patients (mean age: 54(15) years; mean pulmonary arterial pressure: 42(12) mmHg) completed two cycling exercise tests (incremental cardiopulmonary cycling exercise test (CPET) and CET) and three field tests (ISWT, ESWT and six-minute walk test (6MWT)). Physiological parameters were continuously monitored using the same portable telemetric device.

**Results:**

Peak oxygen consumption (VO_2peak_) was similar amongst the five exercise tests (p = 0.90 by ANOVA). Walking distance correlated markedly with the VO_2peak_ reached during field tests, especially when weight was taken into account. At 100% exercise, most physiological parameters were similar between incremental and endurance tests. However, the trends overtime differed. In the incremental tests, slopes for these parameters rose steadily over the entire duration of the tests, whereas in the endurance tests, slopes rose sharply from baseline to 25% of maximum exercise at which point they appeared far less steep until test end. Moreover, cycling exercise tests induced higher respiratory exchange ratio, ventilatory demand and enhanced leg fatigue measured subjectively and objectively.

**Conclusion:**

Endurance tests induce a maximal physiological demand in PAH. Differences in peak respiratory response during exercise are related to the modality (cycling vs. walking) rather than the progression (endurance vs. incremental) of the exercise tests.

## Introduction

Pulmonary arterial hypertension (PAH) is characterized by a progressive increase in pulmonary vascular resistance leading to altered gas exchange, right heart failure and ultimately to patients' death [1]. Numerous exercise abnormalities have been described in PAH. These include considerable reduction of peak oxygen consumption (VO_2peak_), oxygen pulse (VO_2_/HR) and end-tidal carbon dioxide partial pressure (P_ET_CO_2_), abnormal increase in the ventilatory equivalent for carbon dioxide (V_E_/VCO_2_), exercise-induced hypoxemia and early anaerobic threshold [2]–[4]. These abnormalities have been attributed to decreased cardiac output, underperfused alveoli caused by the remodeled and constricted small pulmonary arteries, hyperventilation as well as respiratory and peripheral muscle dysfunction [4]–[7].

Exercise capacity is very meaningful in PAH as it correlates with survival and functional status [8]–[9]. As a result, exercise capacity has been the primary outcome measure in the majority of the recent clinical trials in PAH [10]. While incremental cardiopulmonary cycling exercise test (CPET) provides comprehensive assessment of the cardiac and respiratory adaptation during exercise [9], [11], exercise capacity in PAH is most commonly assessed using the six-minute walk test (6MWT). Following the paradigm set by other chronic conditions such as chronic obstructive pulmonary disease (COPD) in which endurance tests are more sensitive to clinical changes following therapeutic intervention than the CPET and 6MWT [12]–[15], endurance tests including the endurance shuttle walk test (ESWT) and the cycle endurance test (CET) have been proposed in PAH. Whether these tests induce clinically relevant and similar cardiorespiratory response compared to exercise tests currently used in PAH remains unknown.

The objective of this study was to compare the physiological response during endurance (ESWT and CET) and incremental (incremental shuttle walk test (ISWT) and CPET) exercise tests. We tested the hypothesis that endurance tests would induce a near-maximal physiological demand comparable to incremental tests. We also hypothesized that differences in respiratory and skeletal muscle responses during exercise would be related to the modality (cycling vs. walking) rather than the progression (endurance vs. incremental) of the exercise test.

## Methods

### Ethics statement

The institutional ethics committee (Comité d'éthique de la recherche de l'Institut universitaire de cardiologie et de pneumologie de Québec, protocol number: CÉR 20414) approved the research protocol and all patients gave written consent prior to study enrolment.

### Subjects

PAH patients were recruited at the Institut universitaire de cardiologie et de pneumologie de Québec. The PAH diagnosis was made according to recent guidelines [16]. Only patients with no change in their PAH therapies and in stable clinical condition over the last 4 months were eligible. Exclusion criteria were as follow: (1) recent syncope or World Health Organization (WHO) functional class IV [17]; (2) left ventricular ejection fraction <40%; (3) significant restrictive (more than minimal lung fibrosis on CT scan or total lung capacity <70% of predicted) or obstructive (FEV_1_/FVC<70%) lung disease.

### Study design

These measures were obtained as part of a controlled trial evaluating the test-retest reliability of the CET, ESWT and 6MWT [18]. Within two weeks, PAH patients performed two different incremental exercise tests (CPET and ISWT), two constant work rate exercise tests (CET and ESWT) and a 6MWT. Oxygen therapy was not required for any patient during any exercise test.

### Incremental exercise tests

Incremental exercise tests were performed on two different days. Standardized instructions asking patients to exercise up to symptom limitation were given prior each exercise test and standardized encouragements were provided throughout the tests.

At visit 1, a **CPET** was performed on an electrically braked ergocycle (Corival, Lode B.V., Groningen, The Netherlands) [19]. After 3 minutes of rest and 1 minute of unloaded pedaling, patients exercised using a progressive RAMP protocol until exhaustion. Patients were asked to pedal at a minimum rate of 60 rpm and increments varied from 5 to 20 watts/min for target exercise duration between 8 to 12 minutes. After one hour of rest, a practice CET was performed.

At visit 2, an **ISWT** was performed in an enclosed corridor on a flat 10 meter-long course delimited by two cones positioned 0.5 meters from either end [20]. Patients had to follow the rhythm dictated by an audio signal. The initial walking speed was set at 0.5 m·s^−1^ for all patients independently from their age or functional class. Subsequently, the walking speed automatically increased every minute by 0.17 m·s^−1^ until exhaustion. After one hour of rest, a practice ESWT was also performed on the same flat 10 meter-long course [21].

### Constant work rate exercise tests and 6MWT

At visit 3, patients performed two constant work rate exercise tests and a 6MWT, with a resting period of two hours between the tests. To minimize any confounding effect based on test sequence, the order of the tests was randomized using the Latin Square design. While standardized instructions asking the patients to exercise as long as possible during the constant work rate exercises or to cover the longest distance possible during the 6MWT were given prior to each exercise test, no verbal encouragements were made during these exercises [22]. After 5 min of rest, the **CET** was initiated with 1 min of unloaded pedaling before the workload was set at 80% of peak workload achieved during the CPET. Patients were told to pedal at a minimum rate of 60 rpm. The CET time was defined as the total exercise duration until exhaustion excluding unloaded pedaling. The **ESWT** was performed on the same flat 10 meter-long course as for the ISWT. This test included a 1.5 min warm-up period. The walking speed was then set at 85% of the peak walking speed achieved during the ISWT [21]. The rhythm was dictated by an audio signal at constant intervals. The ESWT time was defined as the total exercise duration until exhaustion excluding the warm-up period. The **6MWT** was performed according to the *American Thoracic Society* recommendations [23], and individual results were compared to predicted values [24].

### Physiological monitoring and measurements

Cardiac 12-lead ECG, breath-by-breath respiratory parameters and pulse O_2_ saturation (SpO_2_) were continuously monitored using the same portable telemetric device (Oxycon Mobile, Viasys Healthcare, Hoechberg, Germany) for all exercise tests. The patients were wearing a facemask. The system had a dead space of 30 ml. The transmitting device was composed of two units equipped with O_2_ and CO_2_ analysers. Altogether, the system weighted 950 grams including the lithium battery. Patients wore a shoulder belt system to carry the telemetric device while walking or cycling. The receiving unit was connected to a portable computer. Both volume and gas calibration were made systematically before each exercise test. Cardiac and respiratory parameters were analyzed averaging five of seven breaths for every relative time. The ratio of inspiratory time to total breath duration (Ti/Ttot) and the ratio of tidal volume on inspiratory time (V_T_/Ti) were used as measurements of respiratory timing and inspiratory drive respectively. The anaerobic threshold was assessed using the V-slope method [19]. The V_E_/VCO_2_ slope from rest to the anaerobic threshold was assessed for CPET and ISWT.

Dyspnea and leg fatigue were assessed at the end of each test using a 10-point modified Borg scale [25]. The reasons for stopping exercise were categorized into leg fatigue, dyspnea, general fatigue and inability to maintain the imposed cadence. For field exercise tests, the work of walking was also estimated using the product of the walking distance by body weight [26], assuming a constant velocity during the 6MWT [27]–[28]. In order to assess the objective fatigue of the *vastus lateralis*, both nonvolitional and volitional strengths of the quadriceps were evaluated before and after each constant work rate tests, as previously described [5]. A minimum of 3 sets of maximal voluntary contraction (MVC) and potentiated twitches (TWq) measurements were performed separated by a 1-minute resting period. Reported values for the MVC and TWq were the mean of the three strongest contractions with less than 5% variability. Muscle fatigue was defined as a post-exercise drop in TWq≥15% from baseline [29].

### Statistical analysis

A longitudinal mixed model was used to compare the time course of the various exercise parameters during exercise. For getting a general idea of the trend among the five metabolic levels including baseline, 25%, 50%, 75% and 100% of the total exercise duration, separate smooth fitted curves for each exercise test were done. The data exploration and graphical representation of each subject suggested a piece-wise linear model with a linear increase over the first 25% of the total exercise and then a change in slope with a lower increase afterwards, especially for the CET, the ESWT and the 6MWT. For each outcome variable, two experimental factors were designed including the exercise test (random order) and the relative of the total exercise duration (baseline, 25%, 50%, 75% and 100%). The latter was analyzed as a repeated-measure factor with the use of an autoregressive covariance matrix as correlation decreases as exercise duration between observations increases. A random intercept and random slopes for exercise duration from baseline to 25% and 25% to 100% were considered as subjects have higher or lower intercepts and steeper or shallower slopes over exercise duration. The empirical best linear unbiased predictors (EBLUPs) fulfilled the normality assumption. The interactions between “Exercise test” and the slopes were considered. Missing values were not imputed. The residual maximum likelihood was used as the method of estimation and the Kenward–Roger method was used to estimate denominator degrees of freedom for the test of fixed effect. The univariate normality assumption was verified with the Shapiro-Wilk tests on the error distribution from the Cholesky factorization of the statistical model. The Brown and Forsythe's variation of Levene's test statistic was used to verify the homogeneity of variances. All variables were log-transformed to stabilize variances. The results were considered significant with p-values≤0.05. End-exercise parameters for each exercise modality were compared using ANOVA. Pearson correlation was performed to examine the relationship between workload (cycling tests), as well as distance and work of walking (field tests) and VO_2peak_ reached during each test. Data are presented as mean (SD) or mean (SE) when specified. All analyses were conducted using the statistical packages R v3.0.2 (R Foundation for Statistical Computing, Vienna, Austria.) and SAS v9.4 (SAS Institute Inc, Cary, NC, U.S.A.).

## Results

### Patients' characteristics

Twenty-one PAH patients completed all exercise tests without complication. Baseline characteristics are shown in **Table 1**. No adverse events were observed during exercise tests.

**Table 1:** Patients' characteristics (n = 21).

**PAH Type**	
IPAH	n = 9
PAH-Her	n = 1
PAH-CTD	n = 9
PAH-CHD*	n = 2
**Sex** (F/M)	(17/4)
**Age** (years)	54 (15)
**BMI** (kg·m^−2^)	27 (5)
**WHO Functional Class** (II/III)	(15/6)
**Pulmonary Hemodynamics** **	
RAP (mmHg)	6.6 (3.5)
mPAP (mmHg)	42 (12)
PCWP (mmHg)	11 (3)
CI (l/min·m^2^)	3.0 (0.6)
PVR (WU·m^2^)	6.6 (3.1)
**6MWD** (m)	447 (96)
**6MWD** (% predicted)	83 (17)

Values are n or mean (SD).

* Includes two patients with Eisenmenger physiology related to persistent arterial canal and ventricular septal defect.

** Right heart catheterization performed <6 months was used to describe hemodynamic severity.

*Table legend: PAH, Pulmonary arterial hypertension; IPAH, Idiopathic PAH; PAH-Her, Heritable PAH; PAH-CTD, PAH associated with connective tissue disease; PAH-CHD, PAH associated with congenital heat disease; BMI, Body mass index; WHO, World Health Organization; RAP, Right atrial pressure; mPAP, Mean pulmonary artery pressure; PCWP, Pre-capillary wedge pressure; CI, Cardiac index; PVRi, Pulmonary vascular resistance index; 6MWD, six-minute walk distance.*

### Overall physiological response induced by the various exercise testing modalities

**Figure 1:**
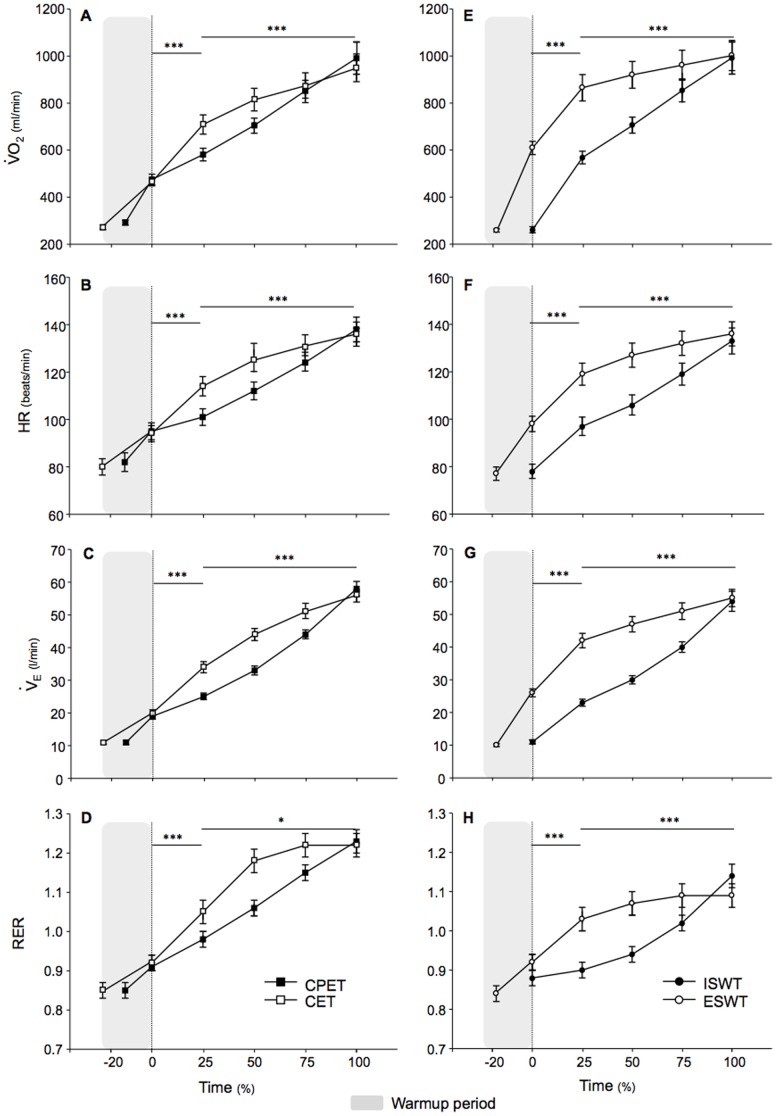
Comparison of the physiological parameters between incremental and constant work rate exercise tests. At 100% exercise, Oxygen consumption (VO_2_), heart rate (HR), minute ventilation (V_E_) and respiratory exchange ratio (RER) were similar between incremental and endurance tests. However, slopes for these parameters rose steadily over the entire duration of the incremental cardiopulmonary exercise test (CPET) and the incremental shuttle walk test (ISWT), whereas their slopes rose sharply from baseline to 25% of maximum exercise at which point they appeared far less steep until test end for the cycle endurance test (CET) and the endurance shuttle walk test (ESWT). Mean (SE) values of physiological parameters are expressed at the same relative time (e.g. 0%, 25%, 50%, 75% and 100%) from the maximal exercise duration. The shaded zone represents the initial warm-up period of the CPET, the CET and the ESWT. *p<0.05; **p<0.01; ***p<0.001 for the comparison of the slopes of each parameter from baseline to 25% of exercise duration, and from 25% to 100% (test end).

Cardiac and ventilatory responses for each exercise test are described in **Table 2** and **Figures 1** **–** **3**, respectively. At 100% exercise, VO_2peak_ was similar for all exercise modalities (p = 0.90 by ANOVA). As expected, the slope of the physiological parameters during the tests significantly differed between exercise modalities (**Figure 1**). In the incremental tests, slopes for oxygen consumption, heart rate (HR), minute ventilation (V_E_), respiratory exchange ratio (RER), as well as carbon dioxide output and V_E_/VCO_2_ (data not shown) rose steadily over the entire duration of the tests. Conversely, in the endurance tests, slopes rose sharply from baseline to 25% of maximum exercise (all p≤0.001 compared to the slopes of incremental tests using the longitudinal mixed model), at which point they appeared far less steep until test end (all p<0.05 compared to the slopes of incremental tests using the longitudinal mixed model). The work rate achieved during CPET and CET significantly correlated with VO_2peak_ reached during these tests (R^2^ = 0.62 and 0.74, respectively, both p<0.01). The mean 6MWT distance was 447(96) meters, as compared to 384(87) and 460(258) meters for the ISWT and ESWT respectively. While the distance walked during the 6MWT, ISWT and ESWT correlated with the VO_2peak_ reached during these tests (R^2^ = 0.25, 0.41 and 0.33, all p<0.01), this correlation was markedly increased when the work of walking was taken into account (R^2^ = 0.75, 0.75 and 0.47, all p<0.01) or when the distance walked during the 6MWT and the ISWT was correlated with the VO_2peak_/kg reached during these tests (R^2^ = 0.67 and 0.75, all p<0.01). Note that these adjustments minimally influenced these correlations for the ESWT (0.47 and 0.20, p<0.01, for the work of walking and the VO_2peak_/kg).

**Figure 2:**
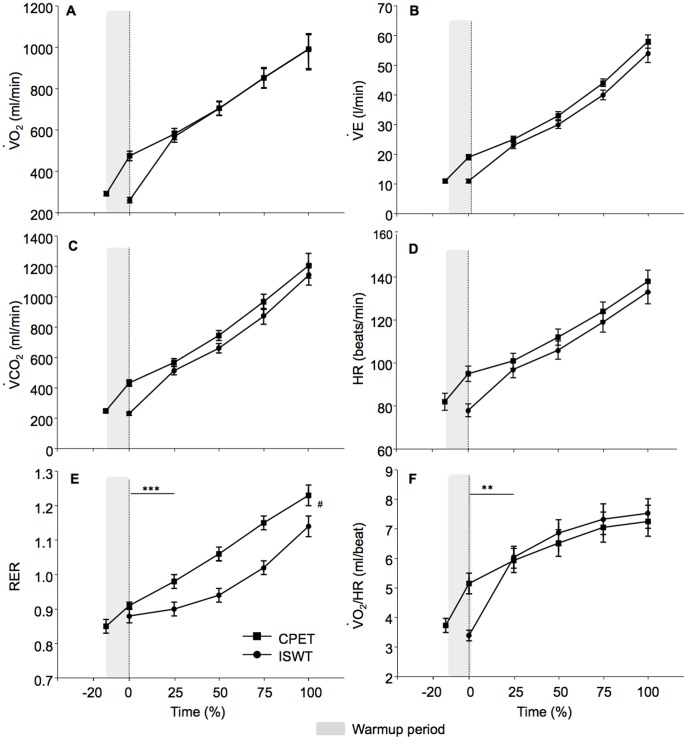
Comparison of the physiological parameters between cycling and walking incremental exercise tests. Physiological response during the cardiopulmonary exercise test (CPET) and the incremental shuttle walk test (ISWT) at the same relative time (e.g. 0%, 25%, 50%, 75% and 100%) from the maximal exercise duration. The CET was characterized by increased respiratory exchange ratio (RER) compared to the ISWT. The shaded zone represents the initial warm-up period of the CPET. Values are means (SE). *p<0.05; **p<0.01; ***p<0.001 for the comparison of the slopes of each parameter from baseline to 25% of exercise duration, and from 25% to 100% (test end). ^#^p<0.05 for end-exercise value compared by ANOVA. *Figure legend:* VO_2_, *Oxygen consumption;* VCO_2_, *Carbon dioxide output; RER, Respiratory exchange ratio;* V_E_, *Minute ventilation; HR, Heart rate;* VO_2_ */HR, Oxygen pulse; CPET, Cardiopulmonary exercise test; ISWT, Incremental shuttle walk test*.

**Figure 3:**
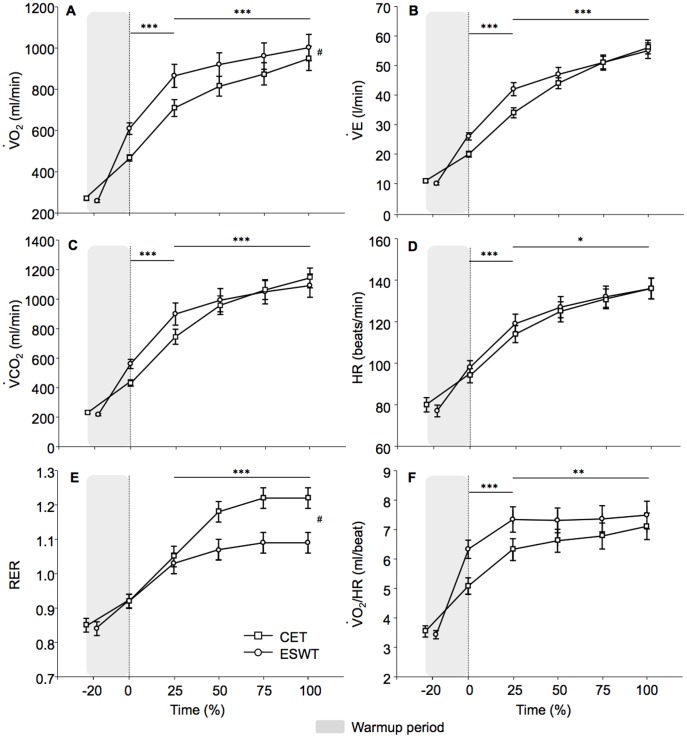
Comparison of the physiological parameters between cycling and walking constant work rate exercise tests. Physiological response during the cycle endurance test (CET) and the endurance shuttle walk test (ESWT) at the same relative time (e.g. 0%, 25%, 50%, 75% and 100%) from maximal exercise duration. The ESWT was characterized by a higher oxygen consumption (VO_2_) and a lower respiratory exchange ratio (RER) throughout the exercise. Conversely, carbon dioxide output (VCO_2_), minute ventilation (V_E_) and oxygen pulse (VO_2_/*HR*) slopes were slightly steeper in the early phase of the CET (from baseline to 25% of exercise duration), to end up with similar end-exercise values. Similarly, heart rate (HR) slopes, although statistically significant, were virtually the same during the CET and the ESWT. The shaded zone represents the initial warm-up period of the ESWT. ^#^p≤0.01 between CET and ESWT. Values are means (SE). *p<0.05; **p<0.01; ***p<0.001 for the comparison of the slopes of each parameter from baseline to 25% of exercise duration, and from 25% to 100% (test end).

**Table 2:** Ventilatory parameters measured at the same relative time during incremental and constant work rate exercise tests as well as the 6MWT (n = 21).

	Incremental exercise tests		Constant work rate exercise tests		6MWT
	CPET	ISWT	CET	ESWT	
BF					
Baseline	19 (4)	19 (5)	19 (5)	20 (4)	20 (5)
25%	25 (7)	25 (6)	27 (3)	33 (6)	29 (5)
50%	29 (7)	29 (6)	32 (8)	37 (7)	33 (6)
75%	33 (6)	32 (6)	37 (7)	38 (6)	35 (6)
100%	40 (7)	39 (7)	40 (7)	40 (7)	37 (8)
V_T_					
Baseline	0.6 (0.1)	0.6 (0.1)	0.6 (0.1)	0.6 (0.1)	0.6 (0.2)
25%	1.1 (0.3)	1.0 (0.2)	1.2 (0.4)	1.3 (0.3)	1.3 (0.3)
50%	1.2 (0.3)	1.1 (0.2)	1.4 (0.4)	1.4 (0.3)	1.3 (0.3)
75%	1.4 (0.3)	1.3 (0.3)	1.4 (0.4)	1.4 (0.3)	1.4 (0.3)
100%	1.5 (0.3)	1.4 (0.3)*	1.4 (0.3)	1.4 (0.3)	1.4 (0.3)
V_T_/Ti (L·sec^1^)					
Baseline	0.53 (0.13)	0.52 (0.16)	0.58 (0.18)	0.51 (0.10)	0.53 (0.15)
25%	1.09 (0.17)	0.95 (0.19)	1.34 (0.29)	1.59 (0.37)	1.38 (0.32)
50%	1.37 (0.22)	1.19 (0.19)	1.66 (0.30)	1.78 (0.39)	1.58 (0.36)
75%	1.68 (0.26)	1.55 (0.26)	1.87 (0.36)	1.85 (0.46)	1.67 (0.35)
100%	2.06 (0.34)	1.91 (0.41)	2.01 (0.38)	1.92 (0.42)	1.80 (0.29)
Ti/Ttot (%)					
Baseline	38 (5)	36 (8)	36 (6)	39 (7)	38 (9)
25%	40 (5)	42 (4)	43 (5)	45 (5)	46 (3)
50%	42 (5)	44 (4)	45 (3)	46 (3)	45 (5)
75%	44 (4)	44 (5)	45 (5)	46 (4)	46 (4)
100%	45 (4)	47 (4)	44 (5)	45 (5)	45 (4)
V_E_/VCO_2_					
Baseline	45 (9)	48 (9)	47 (9)	49 (8)	47 (9)
25%	45 (9)	46 (10)	47 (11)	49 (13)	49 (14)
50%	46 (11)	47 (12)	49 (12)	51 (15)	49 (15)
75%	48 (12)	48 (14)	50 (13)	52 (16)	51 (16)
100%	51 (13)	50 (15)	52 (13)	53 (17)	51 (16)
P_ET_CO_2_ (kPa)					
Baseline	3.83 (0.57)	3.82 (0.53)	3.74 (0.55)	3.77 (0.61)	3.71 (0.56)
25%	3.72 (0.66)	3.59 (0.63)	3.49 (0.74)	3.32 (0.76)	3.34 (0.81)
50%	3.60 (0.77)	3.52 (0.71)	3.34 (0.80)	3.21 (0.75)	3.30 (0.82)
75%	3.45 (0.81)	3.44 (0.80)	3.25 (0.83)	3.14 (0.76)	3.25 (0.83)
100%	3.24 (0.82)	3.23 (0.85)	3.23 (0.80)	3.08 (0.72)	3.17 (0.80)
SpO_2_ (%)					
Baseline	95 (4)	95 (4)	94 (5)	95 (4)	94 (4)
25%	92 (5)	91 (5)	89 (7)	88 (6)	89 (5)
50%	92 (5)	89 (5)	89 (8)	87 (6)	87 (6)
75%	90 (5)	88 (5)	88 (8)	86 (7)	87 (6)
100%	89 (6)	87 (6)	87 (8)	85 (7)	85 (7)
Borg (Leg fatigue)	6 (2)	4 (3)*	6 (2)	5 (2)	5 (2)
Borg (dyspnea)	7 (2)	6 (2)	6 (2)	6 (2)	5 (2)

Values are mean (SD).

*p≤0.05 vs. CPET.

*Table legend: CPET, Cardiopulmonary exercise test; ISWT, Incremental shuttle walk test; CET, Cycle endurance test; ESWT, Endurance shuttle walk test; 6MWT, Six-minute walk test;* V_E_, *Minute ventilation; BF, Breath frequency; V_T_, Tidal volume; Ti, Inspiratory duration; Ttot, Total breath duration;* V_E_/VCO_2_, *Ventilatory equivalent for carbon dioxide; P_ET_CO_2_, End-tidal carbon dioxide partial pressure; SpO_2_, Oxygen saturation by pulse oximetry.*

### Cycling (CPET) versus field walking (ISWT) incremental exercise tests (Table 2, Figure 2)

The maximal workload achieved during CPET was 69(25) watts, with mean increments of 9(2) watts·min^−1^, whereas the maximal walking velocity during ISWT was 1.42(0.28) m·sec^−1^. The mean CPET duration was significantly longer than that of the ISWT (465(124) vs. 370(85) sec, p≤0.01). The slopes and peak values for VO_2_, V_E_, VCO_2_, HR, RER, VO_2_/HR, V_T_/Ti, Ti/Ttot and P_ET_CO_2_ were similar during CPET and ISWT. Nevertheless, RER was higher during CPET. Exercise-induced desaturation was not significantly different during walking (p = 0.08 at end-exercise). Finally, leg fatigue Borg scores were higher following CPET as compared to ISWT, and the most common reason for stopping exercise was different between the two tests as leg fatigue (n = 10/21) was the main limiting factor for CPET as compared to 4/21 for ISWT (p = 0.01).

### Cycling (CET) versus field walking (ESWT) constant work rate exercise tests (Table 2 and Figure 3)

**Figure 4:**
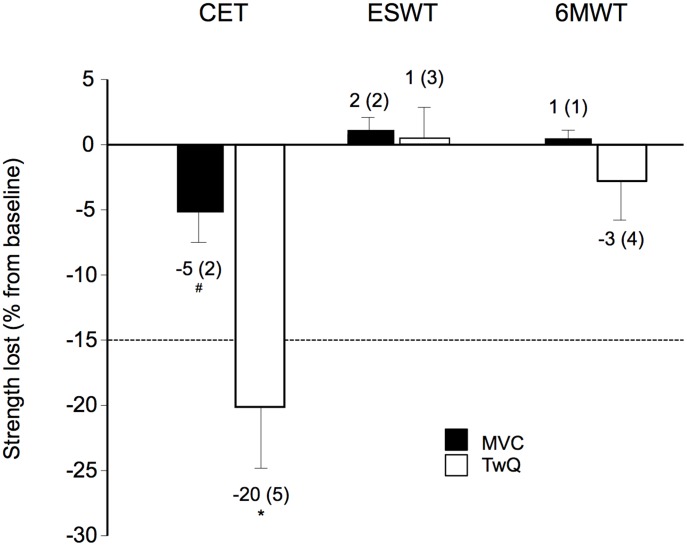
Quadriceps muscle fatigue induced by the constant work rate exercise tests. Relative voluntary and non-volitional quadriceps strengths lost from baseline as assessed by maximal voluntary contraction (MVC) and potentiated twitch (TWq) following the cycle endurance test (CET), the endurance shuttle walk test (ESWT) and the six-minute walk test (6MWT). The CET induced significant quadriceps muscle fatigue defined as a 15% decrease in TWq following exercise (dotted line). Indeed, 12 (57%), 2 (10%) and 3 (14%) patients developed significant quadriceps fatigue following CET, ESWT and 6MWT, respectively. ^*^p≤0.01; ^#^ p≤0.05. Values are mean (SE). *Figure legend: CET, Cycle endurance test; ESWT, Endurance shuttle walk test; 6MWT, Six-minute walk test; MVC, Maximal voluntary contraction; TWq: Potentiated twitches*.

The ESWT duration was longer than CET duration (399(207) versus 248(123) sec, p = 0.01). Although differences in the slopes for VO_2_, V_E_, VCO_2_, HR, RER and VO_2_/HR were statistically significant between CET and ESWT, clinically relevant differences were only observed for end-exercise VO_2_ and RER. Exercise-induced desaturation was also comparable during CET and ESWT (p = 0.13 at end-exercise). Conversely, quadriceps fatigue was enhanced following the CET compared to ESWT (**Figure 4**). Leg fatigue was the main limiting factor in 9/21 and 5/21 patients for the CET and ISWT (p = 0.57).

### Externally paced (ESWT) versus self-paced (6MWT) constant work rate exercise tests

The mean walking velocity (1.24(0.27) vs 1.16(0.23) m·sec^−1^, p = 0.28) and *VO_2_* (1002 (296) vs 953 (258) ml·min^−1^, p = 0.11) were similar for the ESWT and the 6MWT. Nevertheless, ESWT was associated with higher peak VCO_2_ (1092 (364) vs 996 (281) ml·min^−1^, p<0.01), HR (136 (23) vs 128 (21) beat·min^−1^, p<0.01), *V_E_* (55 (12) vs 48 (9) L·min^−1^, p<0.01) and breathing frequency (40 (7) vs 37 (8), p<0.01).

## Discussion

The present study assessed and compared, for the first time, the cardiopulmonary responses of five different exercise testing modalities in patients with PAH. VO_2peak_ were essentially the same amongst the different exercise modalities. The distance walked during field walking exercise tests correlated with the VO_2peak_ achieved during these tests, especially when patients' weight was taken into account. While the pattern of work rate imposition (incremental versus constant work rate) dictated the slopes of the physiological response, the modality of exercise (cycling versus walking) was mainly responsible for differences in end-exercise values for RER, locus of symptom limitation and the degree of quadriceps fatigue.

In this study, VO_2peak_ differed by less than 50 ml among the five different exercise tests. In a previous study, Valli *et al.* [30] described a higher VO_2peak_ during CPET as compared to ISWT in 13 idiopathic PAH patients, although this difference was small in magnitude. Conversely, Deboeck *et al.* [28] documented a higher VO_2_ during the 6MWT compared to CPET in 20 PAH patients. The authors carefully acknowledged that this was possibly related to an underestimation of the aerobic capacity by the CPET as patients were unable to achieve their maximal physiological parameters during this test due to premature lactic acidosis and leg fatigue. Taken together, these results demonstrate that endurance tests are associated with clinically relevant metabolic demand in PAH. Therefore, they may constitute an alternative to incremental cycling test to assess the functional status of patients with PAH.

A major physiological difference between walking and cycling is that the amount of muscles involved is markedly different between these two exercise modalities. In PAH, as in heart failure, the maximum muscle O_2_ extraction is decreased [31] potentially due to muscle capillary rarefaction [7], making VO_2peak_ relatively more muscle-mass dependent than in healthy active subjects. Given the comparable VO_2peak_ for all exercise test, the VO_2_ per muscle unit was likely lower during walking, potentially at a level below the anaerobic threshold. Therefore, the larger muscle mass engaged during walking as compared to cycling could account for lower RER during walking tests. The muscle mecanoreceptors are also more driven by the velocity than by the force deployed [32]–[34]. The differences between cycling speed and walking cadence might thus cause a different ventilatory response. This is also supported by the lower level of quadriceps fatigability and leg fatigue perception during walking than during cycling. These results are also in keeping with previous reports of later onset and less severe lactic acidosis at the same level of load during walking, compared with cycling exercise in other chronic conditions [35]–[36]. These results suggest that the respiratory response during exercise is more related to the exercise modality (cycling vs walking) than its progression (incremental vs. constant). To our knowledge, only one study evaluated the responsiveness of the walking versus cycling tests following a therapeutic intervention in PAH [18]. The responsiveness of CET was lower than the 6MWT and ESWT. Cycling tests might be less susceptible to track a beneficial effect on pulmonary hemodynamics following PAH therapy because of the important drop in non-volitional quadriceps strength as seen in our study.

As the distance walked correlates with the VO_2peak_ of PAH patients, it suggests that field walking exercise tests are representative of the maximal exercise capacity achievable by PAH patients. Interestingly, this correlation between the walking distance and the VO_2peak_ was markedly improved when the work of walking was taken into account, especially for the 6MWT and the ISWT for which the walking distance is directly influenced by the walking velocity. As suggested by Chuang *et al.* [26], the work of walking on a flat course equals the product of distance and weight, assuming a constant walking velocity. However, this estimation does not consider the accelerations and decelerations occurring during the walking tests. This may have led to an underestimation of the work of walking during shuttle walking tests where the 10 meter-long course imposes more acceleration/deceleration than the 30 meter-long course of the 6MWT, and may explain the differences in the physiological response observed between the 6MWT and the ESWT. Whether the work of walking would increase the discriminative capacity of the walking tests in PAH compared to the distance alone also remains to be confirmed.

Potential limitations of our study should be discussed. First, patients had to complete three exercise tests during the same day. This was done to minimize the number of study visits and to accommodate patients coming from remote areas. This could have precluded the achievement of maximal capacity in some tests due to patients' fatigue. Nevertheless, the VO_2peak_ achieved during the two constant work rate exercise tests and the 6MWT were not different than the one achieved during the incremental tests while patients had not previously participated to any forms of exercise. Moreover, the exercise tests were separated by 2 hours and the order of the tests for each subject was randomly determined using the Latin Square design to minimize any confounding effect based on test sequence. Because of technical difficulties in arterial sampling during walking exercise tests, blood gases were not measured. This would have been of interest to accurately quantify dead-space ventilation and to provide indirect information (through blood lactate) about muscle metabolism during exercise tests. Finally, the effect of carrying the telemetric device during walking compared to cycling tests was likely to be trivial given its low weight.

The 6MWT is typically considered as a safe procedure even when performed without detailed cardiopulmonary monitoring. Here, we show that the peak VO_2_ and heart rate responses achieved during a 6MWT represent ≈95% of that of an incremental cycling exercise test, suggesting that this test may induce more physiological stress than previously thought. Despite this, no adverse effects were noted in the present investigation in which a total of 105 exercise tests were performed. This is consistent with previous studies [4], [18], [37] in showing that exercise testing procedure are generally safe in class II/III PAH patients. Our findings may have implications for the choice of exercise tests to be used to assess the efficacy of a given intervention. Considering that cycling tests are more demanding on the lower limb muscle and quadriceps fatigue is more common during cycling, this exercise modality may be preferable to assess interventions such as exercise training whose aim is to improve muscle function. Conversely, cycling tests might be less susceptible to track a beneficial effect on pulmonary hemodynamics following PAH specific therapy, as previously suggested [18]. Similarly, walking tests may be more useful to assess the potential exercise-enhancing effect of oxygen therapy because exercise-induced O_2_ desaturation is more common with this exercise testing modality.

## Conclusions

Despite a similar physiological demand in terms of VO_2peak_, the modality of exercise test was mainly responsible for the different RER, locus of symptom limitation and quadriceps fatigability. The distance reached during field walking tests correlates with the VO_2peak_ achieved during cycling tests and therefore reflects individual exercise capacity in PAH. As it was proposed in COPD, field walking exercise tests might be of clinical interest in PAH. Whether they might be used as an alternative to the 6MWT for tracking beneficial clinical changes following therapy and to assess exercise-induced desaturation remains to be confirmed in PAH.

## References

[1] Rubin LJ (1997) Primary pulmonary hypertension. NEnglJMed 336: 111–117. 10.1056/NEJM199701093360207 8988890

[2] D'Alonzo GE, Gianotti LA, Pohil RL, Reagle RR, DuRee SL, et al (1987) Comparison of progressive exercise performance of normal subjects and patients with primary pulmonary hypertension. Chest 92: 57–62. 3109813 10.1378/chest.92.1.57

[3] Riley MS, Porszasz J, Engelen MP, Brundage BH, Wasserman K (2000) Gas exchange responses to continuous incremental cycle ergometry exercise in primary pulmonary hypertension in humans. EurJApplPhysiol 83: 63–70. 10.1007/s004210000240 11072775

[4] Sun XG, Hansen JE, Oudiz RJ, Wasserman K (2001) Exercise pathophysiology in patients with primary pulmonary hypertension. Circulation 104: 429–435. 11468205 10.1161/hc2901.093198

[5] Mainguy V, Maltais F, Saey D, Gagnon P, Martel S, et al (2010) Peripheral muscle dysfunction in idiopathic pulmonary arterial hypertension. Thorax 65: 113–117. 19720606 10.1136/thx.2009.117168

[6] Meyer FJ, Lossnitzer D, Kristen AV, Schoene AM, Kubler W, et al (2005) Respiratory muscle dysfunction in idiopathic pulmonary arterial hypertension. EurRespirJ 25: 125–130. 10.1183/09031936.04.00095804 15640333

[7] Potus F, Malenfant S, Graydon C, Mainguy V, Tremblay E, et al (2014) Impaired Angiogenesis and Peripheral Muscle Microcirculation Loss Contributes to Exercise Intolerance in Pulmonary Arterial Hypertension. Am J Respir Crit Care Med Epub ahead of print. 10.1164/rccm.201402-0383OC 24977625

[8] Miyamoto S, Nagaya N, Satoh T, Kyotani S, Sakamaki F, et al (2000) Clinical correlates and prognostic significance of six-minute walk test in patients with primary pulmonary hypertension. Comparison with cardiopulmonary exercise testing. AmJRespirCrit Care Med 161: 487–492. 10.1164/ajrccm.161.2.9906015 10673190

[9] Wensel R, Opitz CF, Anker SD, Winkler J, Hoffken G, et al (2002) Assessment of survival in patients with primary pulmonary hypertension: importance of cardiopulmonary exercise testing. Circulation 106: 319–324. 12119247 10.1161/01.cir.0000022687.18568.2a

[10] Galie N, Manes A, Negro L, Palazzini M, Bacchi-Reggiani ML, et al (2009) A meta-analysis of randomized controlled trials in pulmonary arterial hypertension. EurHeart J 30: 394–403. 10.1093/eurheartj/ehp022 PMC2642921 19155250

[11] McGoon M, Gutterman D, Steen V, Barst R, McCrory DC, et al (2004) Screening, early detection, and diagnosis of pulmonary arterial hypertension: ACCP evidence-based clinical practice guidelines. Chest 126: 14S–34S. 15249493 10.1378/chest.126.1_suppl.14S

[12] Dyer CA, Singh SJ, Stockley RA, Sinclair AJ, Hill SL (2002) The incremental shuttle walking test in elderly people with chronic airflow limitation. Thorax 57: 34–38. 11809987 10.1136/thorax.57.1.34 PMC1746182

[13] Eiser N, Willsher D, Dore CJ (2003) Reliability, repeatability and sensitivity to change of externally and self-paced walking tests in COPD patients. Respiratory medicine 97: 407–414. 12693802 10.1053/rmed.2002.1462

[14] Verkindre C, Bart F, Aguilaniu B, Fortin F, Guerin JC, et al (2006) The effect of tiotropium on hyperinflation and exercise capacity in chronic obstructive pulmonary disease. Respiration; international review of thoracic diseases 73: 420–427. 16484769 10.1159/000089655

[15] Eaton T, Young P, Nicol K, Kolbe J (2006) The endurance shuttle walking test: a responsive measure in pulmonary rehabilitation for COPD patients. ChronRespirDis 3: 3–9. 10.1191/1479972306cd077oa 16509172

[16] Simonneau G, Gatzoulis MA, Adatia I, Celermajer D, Denton C, et al (2013) Updated clinical classification of pulmonary hypertension. J Am Coll Cardiol 62: D34–41. 24355639 10.1016/j.jacc.2013.10.029

[17] Humbert M, Sitbon O, Simonneau G (2004) Treatment of pulmonary arterial hypertension. NEnglJMed 351: 1425–1436. 10.1056/NEJMra040291 15459304

[18] Mainguy V, Malenfant S, Neyron AS, Bonnet S, Maltais F, et al (2013) Repeatability and responsiveness of exercise tests in pulmonary arterial hypertension. Eur Respir J 42: 425–434. 23100508 10.1183/09031936.00107012

[19] ATS/ACCP Statement on cardiopulmonary exercise testing. AmJRespirCrit Care Med 167: 211–277. 10.1164/rccm.167.2.211 12524257

[20] Singh SJ, Morgan MD, Scott S, Walters D, Hardman AE (1992) Development of a shuttle walking test of disability in patients with chronic airways obstruction. Thorax 47: 1019–1024. 1494764 10.1136/thx.47.12.1019 PMC1021093

[21] Revill SM, Morgan MD, Singh SJ, Williams J, Hardman AE (1999) The endurance shuttle walk: a new field test for the assessment of endurance capacity in chronic obstructive pulmonary disease. Thorax 54: 213–222. 10325896 10.1136/thx.54.3.213 PMC1745445

[22] Guyatt GH, Pugsley SO, Sullivan MJ, Thompson PJ, Berman L, et al (1984) Effect of encouragement on walking test performance. Thorax 39: 818–822. 6505988 10.1136/thx.39.11.818 PMC459930

[23] ATS (2002) ATS statement: guidelines for the six-minute walk test. AmJRespirCrit Care Med 166: 111–117. 10.1164/ajrccm.166.1.at1102 12091180

[24] Enright PL, Sherrill DL (1998) Reference equations for the six-minute walk in healthy adults. Am J RespirCrit Care Med 158: 1384–1387. 10.1164/ajrccm.158.5.9710086 9817683

[25] Borg GA (1982) Psychophysical bases of perceived exertion. MedSciSports Exerc 14: 377–381. 7154893

[26] Chuang ML, Lin IF, Wasserman K (2001) The body weight-walking distance product as related to lung function, anaerobic threshold and peak VO2 in COPD patients. RespirMed 95: 618–626. 10.1053/rmed.2001.1115 11453321

[27] Casas A, Vilaro J, Rabinovich R, Mayer A, Barbera JA, et al (2005) Encouraged 6-min walking test indicates maximum sustainable exercise in COPD patients. Chest 128: 55–61. 16002916 10.1378/chest.128.1.55

[28] Deboeck G, Niset G, Vachiery JL, Moraine JJ, Naeije R (2005) Physiological response to the six-minute walk test in pulmonary arterial hypertension. EurRespirJ 26: 667–672. 10.1183/09031936.05.00031505 16204599

[29] Saey D, Debigare R, LeBlanc P, Mador MJ, Cote CH, et al (2003) Contractile leg fatigue after cycle exercise: a factor limiting exercise in patients with chronic obstructive pulmonary disease. AmJRespirCrit Care Med 168: 425–430. 10.1164/rccm.200208-856OC 12714348

[30] Valli G, Vizza CD, Onorati P, Badagliacca R, Ciuffa R, et al (2007) Pathophysiological adaptations to walking and cycling in primary pulmonary hypertension. EurJApplPhysiol 10.1007/s00421-007-0600-y 17978836

[31] Tolle J, Waxman A, Systrom D (2008) Impaired systemic oxygen extraction at maximum exercise in pulmonary hypertension. MedSciSports Exerc 40: 3–8. 10.1249/mss.0b013e318159d1b8 18091026

[32] Casey K, Duffin J, Kelsey CJ, McAvoy GV (1987) The effect of treadmill speed on ventilation at the start of exercise in man. The Journal of physiology 391: 13–24. 3127577 10.1113/jphysiol.1987.sp016722 PMC1192198

[33] Kelsey CJ, Duffin J (1992) Changes in ventilation in response to ramp changes in treadmill exercise load. European journal of applied physiology and occupational physiology 65: 480–484. 1425657 10.1007/BF00243518

[34] Takano N (1988) Effects of pedal rate on respiratory responses to incremental bicycle work. The Journal of physiology 396: 389–397. 3137329 10.1113/jphysiol.1988.sp016968 PMC1192051

[35] Palange P, Forte S, Onorati P, Manfredi F, Serra P, et al (2000) Ventilatory and metabolic adaptations to walking and cycling in patients with COPD. JApplPhysiol 88: 1715–1720. 10.1152/jappl.2000.88.5.1715 10797134

[36] Page E, Cohen-Solal A, Jondeau G, Douard H, Roul G, et al (1994) Comparison of treadmill and bicycle exercise in patients with chronic heart failure. Chest 106: 1002–1006. 7924466 10.1378/chest.106.4.1002

[37] Groepenhoff H, Vonk-Noordegraaf A, Boonstra A, Spreeuwenberg MD, Postmus PE, et al (2008) Exercise testing to estimate survival in pulmonary hypertension. Med Sci Sports Exerc 40: 1725–1732. 18799981 10.1249/MSS.0b013e31817c92c0

